# Chordin-Like 2: A Possible Therapeutic Target for Gastric Cancer by Affecting Cell Cycle and Proliferation

**DOI:** 10.1155/2022/4607715

**Published:** 2022-11-08

**Authors:** Yuan Zhou, Guangxin Cao, Zhifeng Guan, Cui Mao

**Affiliations:** ^1^Department of Hepatobiliary Surgery, Tumor Hospital Affiliated to Nantong University, Nantong Tumor Hospital, Nantong, China; ^2^Department of General Surgery, Tumor Hospital Affiliated to Nantong University & Nantong Tumor Hospital, Nantong, Jiangsu 226300, China; ^3^Department of Radiotherapy, Tumor Hospital Affiliated to Nantong University, Nantong Tumor Hospital, Nantong, China; ^4^Department of General Surgery, Affiliated Nantong Hospital 3 of Nantong University, Nantong, China

## Abstract

**Purpose:**

This study aimed to examine the role of chordin-like 2 (CHRDL2) in gastric cancer.

**Methods:**

The Gene Expression Omnibus (GEO) and the Cancer Genome Atlas (TCGA) datasets were screened and the differentially expressed gene *CHRDL2* was identified. The CHRDL2 expression was examined in the Human Protein Atlas and TCGA. Clinical data on gastric cancer were evaluated for their association with CHRDL2 by using TCGA and KM-plotter databases. The possible relationship amongst CHRDL2, immune cells, and related genes was investigated via the TIMER database. Enrichment analysis was performed using GO and KEGG pathways to explore the mechanisms.

**Results:**

Screening of databases revealed that CHRDL2 was a differentially expressed gene. An increase in cytoplasmic CHRDL2 expression was found in cancer tissues compared with the surrounding normal tissues. The data, together with those from TCGA and the KM-plotter databases, showed that patients with gastric cancer with high level of CHRDL2 have worse prognosis than those with low expression. A strong correlation was found between CHRDL2 expression and T stage, race, pathological grade, and pathological type according to clinical data analysis. CHRDL2 expression is linked to immune infiltration, as shown by the TIMER database. The data suggested that CHRDL2 plays a pivotal role in the tumor microenvironment of gastric cancer and might help tumor cells evade the immune system. Gene set enrichment analysis showed that CHRDL2 is involved in the chemokine signaling route, the intestinal immune network, the MAPK pathway, cell cycle, and the PI3K-Akt signaling system that are associated with the pathological processes of gastric cancer.

**Conclusion:**

Patients with gastric cancer with decreased CHRDL2 levels have dramatically improved OS, PFS, and PPS. CHRDL2 plays a pivotal role in enabling tumor cell immune evasion in tumor microenvironment, suggesting a function of this gene in the development of gastric cancer and its immune infiltration. Interfering with CHRDL2 may slow down the development of this malignancy by affecting cell cycle and apoptosis pathways.

## 1. Introduction

Gastric cancer is a malignant tumor in the stomach which develops from the epithelium lining the gastric mucosa. Gastric cancer has the fifth highest incidence and third highest fatality rate in the world, causing about 720,000 deaths each year [2]. Because there are no distinctive symptoms that are associated with stomach cancer and the clinical diagnostic rate is low, most patients are discovered at a late stage when the effectiveness of therapy and the overall prognosis are poor [3]. In the treatment of stomach cancer, radical surgical resection is a key, yet the postoperative recurrence rate is high [4]. Adjuvant chemotherapy is often used as a therapeutic option for individuals with advanced gastric cancer who are candidates for surgery [5]. Patients with stomach cancer could benefit greatly from an accurate assessment of their overall condition so that appropriate treatment choices may be made. However, no optimal prognostic biomarkers are currently available for this purpose. Comprehensive molecular characterisation of gastric cancer has been performed by the Cancer Genome Atlas research network and microsatellite instability (MSI) is utilised as classification for genomic subgroups [6]. Existing study has found that oncogenes, tumor suppressor genes, and signaling pathways are related to the occurrence and development of gastric carcinoma. For example, CA72-4 plays an important role in the auxiliary diagnosis of gastric cancer, monitoring dynamic progress and evaluating prognosis. CA72-4 can induce the novel aptamer to react with tumor cells and enhance the efficacy of trastuzumab in HER-2-positive gastric cancer [7]. Claudin-6 is involved in gastric cancer by affecting cell cycle and p53 signaling in gastric cancer [8]. ST3GAL6, a circular RNA, inhibits gastric cancer by activating autophagy through the FOXP2/MET/mTOR axis [9]. However, the molecular pathophysiology of stomach cancer is not well understood and no optimal tumor indicators could be used for early identification and prognosis. Thus, finding tumor markers for early detection and prognosis of gastric cancer is crucial.

Bone morphogenetic proteins (BMPs) are important secreted cytokines and they belong to the TGF-*β* protein family, which could transduce signals through Smad1, Smad5, and Smad8 [10]. Secreted proteins known as TGF-*β* superfamily ligand antagonists (or ETAs) promote tumor formation by inhibiting TGF-*β* signaling [11]. Chordin-like 2 (CHRDL2) acts as a BMP antagonist by blocking the action of BMP at its corresponding cell surface receptors [12, 13]. As revealed by Chen et al. [14] and others, CHRDL2 promotes the growth and spread of osteosarcoma cells by activating the BMP-9/PI3K/AKT signaling pathway. Previous research [15] also indicated that CHRDL2 suppresses p-Smad1/5 by binding to BMP, thereby boosting CRC cell proliferation and blocking apoptosis.

In this study, TCGA database was screened for the differentially expressed genes that are related to gastric cancer prognosis. We identified CHRDL2, which was shown to have a role in predicting survival in patients with gastric cancer. Bioinformatics analysis was conducted to investigate how CHRDL2 affects tumor cell behaviour and its underlying mechanisms.

## 2. Materials and Methods

### 2.1. Dataset Analysis

The GEO database (https://www.ncbi.nlm.nih.gov/geo/query/acc.cgi) was queried to obtain GSE19826, GSE54129, and GSE49051 RNA expression datasets (including tumor and normal tissues). Multiple probes for the same molecule were ignored in favour of the one with the highest signal strength. By drawing a Venn diagram with these three datasets as the intersection, CHRDL2 could be identified as a gene associated with gastric cancer prognosis with the use of the criterion for identifying differentially expressed genes (Ilog2FCI > 1 and *p*  <  0.05).

### 2.2. Tissue-Level CHRDL2 Expression in Human Protein Atlas

The location of CHRDL2 in the body was further determined by searching for its expression in the Human Protein Atlas (https://www.proteinatlas.org/search/HAMP).

### 2.3. CHRDL2 in KM-Plotter and Gastric Cancer Data

Clinical data on gastric cancer in the KM-plotter database (https://kmplot.com/analysis/index.php?p=service) were examined to determine whether CHRDL2 is associated with the prognosis of patients with gastric cancer.

### 2.4. Correlation between CHRDL2 and Immune Cells

A bar graph was built using the TIMER database (https://cistrome.shinyapps.io/timer/) to visualise the association between CHRDL2 expression and different immune cells in gastric cancer.

### 2.5. GO and KEGG Enrichment Analysis

Metascape (https://metascape.org/gp/index.html#/main/step1) was used for functional analysis of differentially expressed genes.

### 2.6. CHRDL2 in TCGA and Gastric Cancer Data

CHRDL2 protein was correlated with the clinical data and outcomes of patients with gastric cancer via analysis of TCGA database.

### 2.7. Statistical Analysis

Data were downloaded using the GEOquery program (version 2.54.1) in R [16], and the limma package (version 3.42.2) [17] was used for statistical analysis and visualisation. The potential biological mechanism of CHRDL2 was investigated using gene set enrichment analysis (GSEA). Statistical analysis was performed on SPSS version 22 (IBM Corporation). The chi-square test was implemented to compare the clinicopathological characteristics of the two cohorts. The Kaplan–Meier method was used to calculate patients' survival times. The log rank test was used to assess statistical significance, and the Spearman correlation coefficient was used to evaluate the strength of their association. The change was statistically significant if *p* value <0.05.

## 3. Results

### 3.1. Increased Expression of CHRDL2 in Gastric Cancer

**Figure 1:**
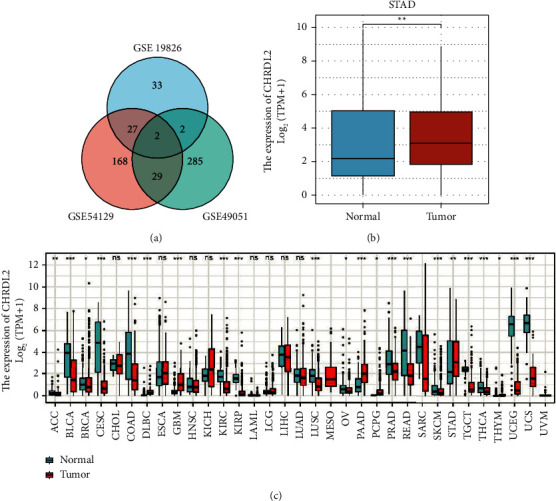
Gastric cancer had increased CHRDL2 expression. (a) The gene Venn diagrams of the three datasets. (b) Pan-cancer CHRDL2 expression. (c) TCGA database included 210 para-cancerous tissues and 414 unpaired stomach cancer tissues, and CHRDL2 was substantially expressed in tumor tissues.

The initial step was to use a Venn diagram to compare the three datasets GSE19826, GSE54129, and GSE49051, and CHRDL2 was identified as a differentially expressed gene (Figure 1(a)). Pan-cancer study revealed that CHRDL2 expression was upregulated in ESCA, GBM, KICH, PAAD, STAD, and other tumor tissues and downregulated in CESC, COAD, KIRC, KIRP, LIHC, READ, and SARC (Figure 1(b)). Analysis of TCGA database showed that CHRDL2 mRNA expression was greater in gastric cancer tumor tissues than in adjacent tissues (Figure 1(b)), consistent with the results of pan-cancer study.

### 3.2. Tissue-Specific Expression of CHRDL2 in Human Protein Atlas

**Figure 2:**
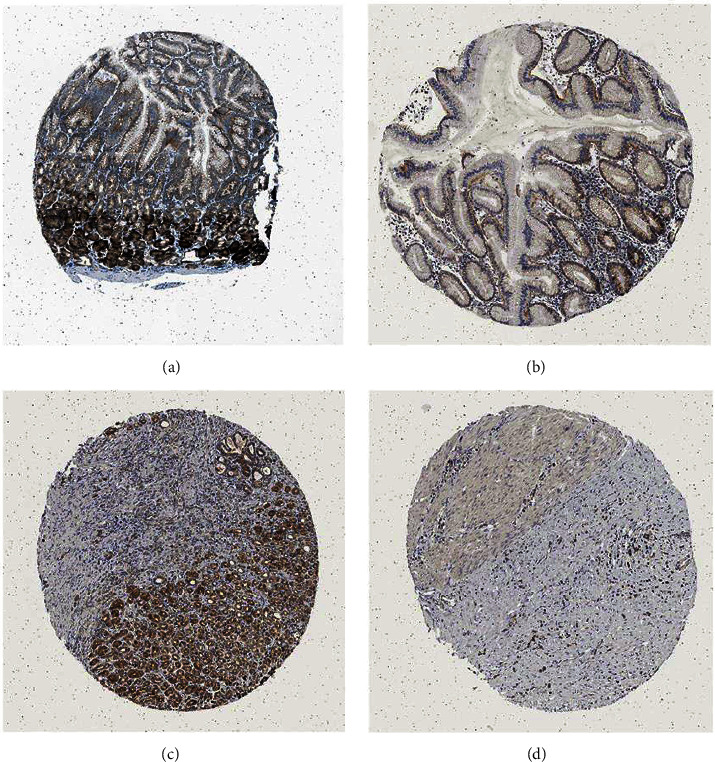
Expression of CHRDL2 at the tissue level in the Human Protein Atlas database. (a) High level of CHRDL2 expression. (b) Modest level of CHRDL2 expression in healthy stomach tissue. (c) High expression of CHRDL2. (d) Low expression of CHRDL2 in gastric cancer tissue according to immunohistochemistry.

CHRDL2 was mostly found in the cytoplasm of cells, as shown through searching its expression in the Human Protein Atlas (Figures 2(a)–2(d)).

### 3.3. Correlation between CHRDL2 in KM-Plotter Database and Prognosis of Gastric Cancer

**Figure 3:**
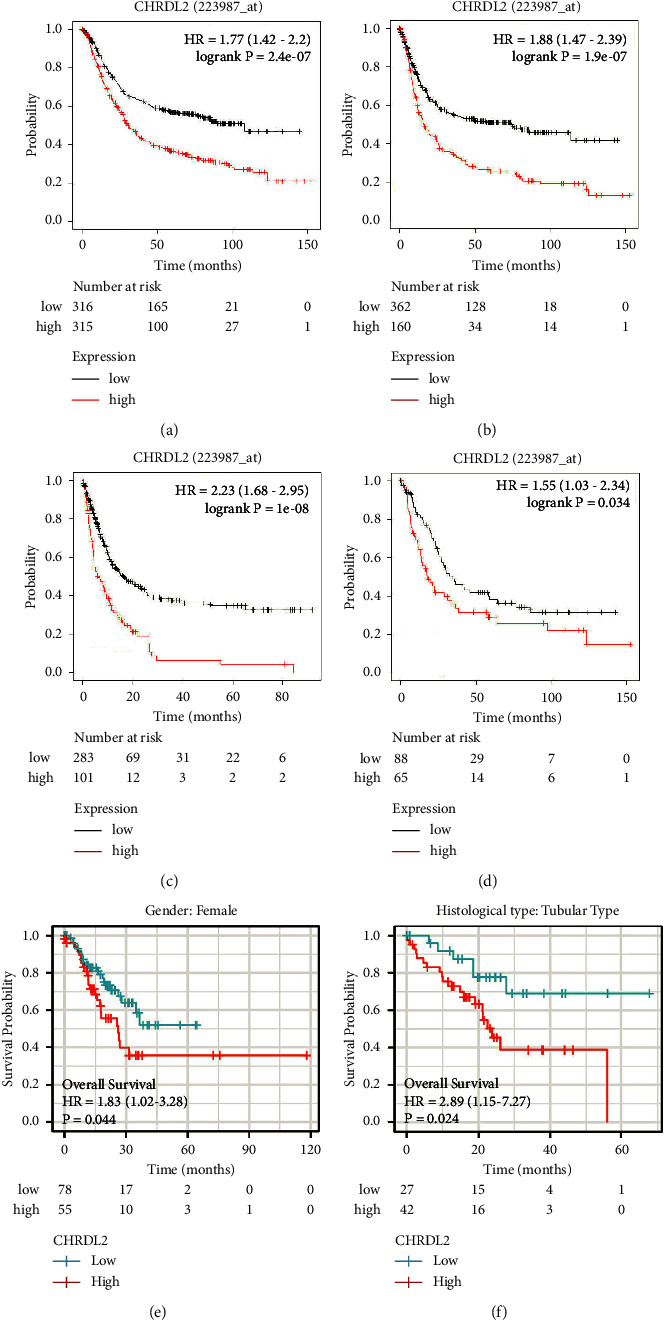
The association of CHRDL2 expression and prognosis in patients with stomach cancer in the KM-plotter database. (a) CHRDL2 and OS in individuals with gastric cancer. (b) CHRDL2 and PFS correlation in patients with gastric cancer. (c) CHRDL2 and PPS correlation in patients with gastric cancer. (d) Correlation between prognosis of patients with HER-2-positive gastric cancer and CHRDL2 expression. (e) The significance of CHRDL2 and prognosis in female gastric cancer patients. (f) Correlation between CHRDL2 expression and prognosis of patients with gastric tubular adenocarcinoma.

The patients with high CHRDL2 expression had worse OS (Figure 3(a)), PFS (Figure 3(b)), and PPS (Figure 3(c)) outcomes than those with low CHRDL2 expression based on the study of the clinical data for gastric cancer in the KM-plotter database (Figure 3(c)). By further stratifying analysis, we found that in HER-2-positive female gastric cancer patients with pathological type of tubular adenocarcinoma, all patients with low expression of CHRDL2 had a better prognosis (Figures 3(d)–3(f)).

### 3.4. Correlation of CHRDL2 and Immune Cells in TIMER

**Figure 4:**
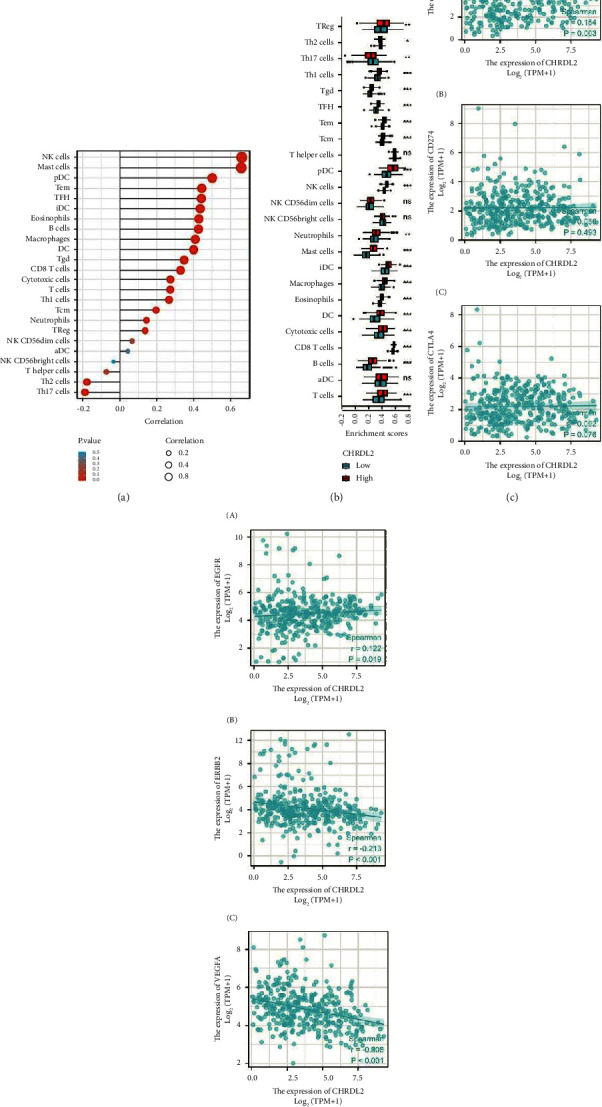
CHRDL2 expression significantly correlated with immune cell infiltration in gastric cancer (a). CHRDL2 expression positively correlated with infiltration of majority of immune cells in the TIMER database (b). CHRDL2 expression in gastric cancer correlated with PDCD1, CD274, and CTLA-4 (c). Scatter plot showing the relationship amongst CHRDL2, EGFR, ERBB2, and VEGFA expression levels in gastric cancer (d).

The TIMER database was utilised to create pie charts contrasting CHRDL2 expression with various immune cell types in gastric cancer. A bulk of invading immune cells, including NK cells, mast cells, macrophages, Th1 cells, B cells, DCs, Tgd, Tcm, and TReg, were found to express CHRDL2 and the expression was linked with disease progression (Figure 4(a)). The infiltration level of most immune cells, including Treg, Th2, Th1, and Th17 cells, was correlated with CHRDL2, and this association was examined to further understand the effect of CHRDL2 on the tumor microenvironment (TME, Figure 4(b)). Additional research revealed a strong association between CHRDL2 expression and PDCD1, a molecule associated with immunological checkpoints, but a non-significant correlation was found between CTLA-4 and CD274 (Figure 4(c)). Considering the specificity of gastric cancer and the current targeted therapy, CHRDL2 and gastric cancer-related therapeutic targets were studied. The study on CHRDL2 suggested that it was highly correlated with EGFR, ERBB2, and VEGFA (*p*  <  0.05, Figure 4(d)), which may serve as a point of reference in the creation of new targeted therapies.

### 3.5. GSEA Results

**Figure 5:**
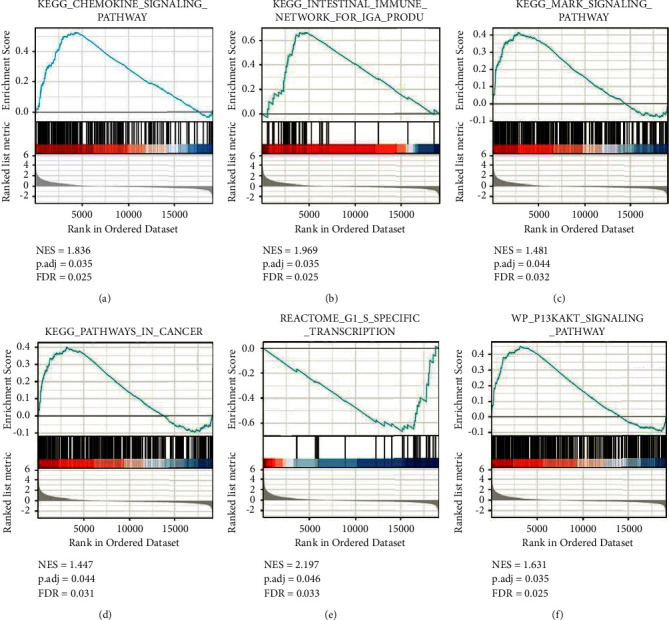
Six possibly relevant and statistically significant pathways.

Six potentially relevant pathways with statistical significance were obtained through GSEA: KEGG_CHEMOKINE_SIGNALING_PATHWAY, KEGG_INTESTINAL_IMMUNE_NETWORK_FOR_IGA_PRODUCTION, KEGG_MAPK_SIGNALING_PATHWAY, KEGG_PATHWAYS_IN_CANCER, REACTOME_G1_S_SPECIFIC_TRANSCRIPTION, and WP_PI3KAKT_SIGNALING_PATHWAY (Figures 5(a)–5(f)).

Therefore, CHRDL2 possibly affects the poor prognosis of gastric cancer by affecting cell cycle and proliferation. This finding should be verified by basic experimental research.

The biological process gene set from MSigDB, known as Gene Ear Biology, was used. A total of 1000 different variations of the random sample could be found. NOM-p stands for nominal *p* value, NES denotes normalised enrichment score, and FDR-q refers to false discovery rate.

### 3.6. CHRDL2 and Clinicopathological Characteristics of Gastric Cancer

Based on the median CHRDL2 expression, TCGA database of patients with gastric cancer was divided into two groups, namely, high and low-CHRDL2 expression groups, to further understand the relationship between CHRDL2 expression and the clinicopathological aspects of patients with gastric cancer. CHRDL2 expression was found to be substantially (*p*  <  0.05) linked with T stage, pathological grade, ethnicity, pathological type, and pathological grade (Table 1).

## 4. Discussion

Gastric cancer is a frequent malignant tumor of the digestive system. According to the most recent global cancer statistics made public by the World Health Organization's International Agency for Research on Cancer, gastric cancer accounted for the fifth highest incidence of malignant tumors worldwide in 2020 [18]. The gradual signs of stomach cancer are easy to be missed, and many patients are diagnosed with the disease at an advanced stage with invasion or metastases. Even if radical resection is performed, 40%–65% of patients experience recurrence or metastasis after surgery, with survival time of less than a year. Inhibiting or activating certain immunological checkpoints [19, 20] is one method in which the current immunotherapy approach focusing on the involvement of the immune milieu may revert the immune escape of malignancies. Clinical trials validating the efficacy of immune checkpoint therapy in treating malignancies have been conducted, and they are available for perusal [21]. Identifying novel sensitive molecular markers and therapeutic targets is essential to enhance the prognosis of patients with gastric cancer.

The expression of CHRDL2 in pan-cancer was firstly identified through GEO database screening for differentially expressed genes. Subsequent bioinformatics and database searches provided an in-depth understanding of CHRDL2's role in cancers. Consistent with the findings of several other studies, the present study showed that CHRDL2 was expressed in ESCA, GBM, and KICH and upregulated in PAAD, STAD, and other tumor tissues. Meanwhile, CHRDL2 expression was downregulated in CESC, COAD, KIRC, KIRP, LIHC, READ, SARC, and other tumor tissues. Finally, CHRDL2's tissue-specific expression was explored using data from the Human Protein Atlas. The results showed that it was mostly cytoplasmic and expressed at greater levels in less differentiated tissues. Patients with gastric cancer who had high expression of CHRDL2 showed worse OS, PFS, and PPS than those whose CHRDL2 expression was low. Patients with HER-2-positive tubular adenocarcinoma of the stomach and low CHRDL2 expression had the best survival rates. Thus, CHRDL2 has a role in stomach cancer development.

Tumor-infiltrating lymphocytes are of special interest because they are an independent predictor of sentinel lymph node status and survival in patients with cancer [22, 23]. The use of public data mining showed that CHRDL2 expression was linked to immune cell infiltration in gastric cancer, and this link was strengthened when a bulk of infiltrating immune cells, such as NK cells, mast cells, macrophages, Th1 cells, B cells, DC, Tgd, Tcm, and Treg cells, were CHRDL2 positive. CHRDL2 expression was also correlated well with the immune checkpoint-related molecule PDCD1. Considering the specificity of gastric cancer and the current targeted therapy, CHRDL2 and gastric cancer-related therapeutic targets were studied. CHRDL2 was found to have good correlation with EGFR, ERBB2, and VEGFA. Finally, six statistically significant related pathways were obtained through GSEA: REACTOME_G1_S_SPECIFIC_TRANSCRIPTION, KEGG_CHEMOKINE_SIGNALING_PATHWAY, KEGG_MAPK_SIGNALING_PATHWAY, WP_PI3KAKT_SIGNALING_PATHWAY, KEGG_PATHWAYS_IN_CANCER, and KEGG_INTESTINAL_IMMUNE_NETWORK_FOR_IGA_PRODUCTION. Previous research [24] indicated that norepinephrine increased pancreatic cancer cell proliferation by activating the P38/MAPK signaling pathway in a *β*-adrenergicreceptor-dependent manner. Through the MAPK pathway, the long non-coding RNA cancer susceptibility candidate 2 suppressed cervical cancer cell replication, invasion, and creation of new blood vessels [25]. PBRM1 could regulate renal cell carcinoma proliferation and cell cycle through the chemokine/chemokine receptor interaction pathway [26]. Strychnine inhibited human lung cancer cells by arresting the cell cycle and proliferation of PC-9 [27]. Therefore, based on these predicted pathway results, CHRDL2 affects the poor prognosis of gastric cancer by affecting cell cycle and proliferation.

## 5. Conclusion

In conclusion, this research demonstrated that CHRDL2 could be a biomarker of gastric cancer disease progression. CHRDL2 was defined as a regulator of cell proliferation, apoptosis induction, and cell cycle progression. Gastric cancer development may be considerably slowed down by interfering CHRDL2 as this protein regulates cell cycle and apoptosis pathways. The results of this study showed a possible mechanism that may be used in the future to produce effective treatments for stomach cancer.

## Figures and Tables

**Table 1:** Correlation of CHRDL2 with clinicopathological parameters in patients with gastric cancer.

Characteristics	Levels	Low expression of CHRDL2	High expression of CHRDL2	*p*
*n*		187	188	
T stage, *n* (%)	T1	16 (4.4%)	3 (0.8%)	0.010
	T2	43 (11.7%)	37 (10.1%)	
	T3	82 (22.3%)	86 (23.4%)	
	T4	43 (11.7%)	57 (15.5%)	

N stage, *n* (%)	N0	63 (17.6%)	48 (13.4%)	0.276
	N1	45 (12.6%)	52 (14.6%)	
	N2	37 (10.4%)	38 (10.6%)	
	N3	32 (9%)	42 (11.8%)	

M stage, *n* (%)	M0	163 (45.9%)	167 (47%)	0.415
	M1	15 (4.2%)	10 (2.8%)	

Pathologic stage, *n* (%)	Stage I	38 (10.8%)	15 (4.3%)	0.002
	Stage II	53 (15.1%)	58 (16.5%)	
	Stage III	64 (18.2%)	86 (24.4%)	
	Stage IV	22 (6.2%)	16 (4.5%)	

Primary therapy outcome, *n* (%)	SD	8 (2.5%)	9 (2.8%)	0.414
	PD	37 (11.7%)	28 (8.8%)	
	CR	109 (34.4%)	122 (38.5%)	
	PR	1 (0.3%)	3 (0.9%)	

Gender, *n* (%)	Male	118 (31.5%)	123 (32.8%)	0.718
	Female	69 (18.4%)	65 (17.3%)	

Race, *n* (%)	Asian	43 (13.3%)	31 (9.6%)	0.014
	White	101 (31.3%)	137 (42.4%)	
	Black or African American	8 (2.5%)	3 (0.9%)	

Histological type, *n* (%)	Diffuse type	26 (7%)	37 (9.9%)	0.010
	Mucinous type	5 (1.3%)	14 (3.7%)	
	Not otherwise specified	105 (28.1%)	102 (27.3%)	
	Papillary type	3 (0.8%)	2 (0.5%)	
	Signet ring type	3 (0.8%)	8 (2.1%)	
	Tubular type	45 (12%)	24 (6.4%)	

Age, *n* (%)	≤65	72 (19.4%)	92 (24.8%)	0.065
	>65	112 (30.2%)	95 (25.6%)	

Residual tumor, *n* (%)	R0	146 (44.4%)	152 (46.2%)	0.296
	R1	8 (2.4%)	7 (2.1%)	
	R2	11 (3.3%)	5 (1.5%)	

Histologic grade, *n* (%)	G1	5 (1.4%)	5 (1.4%)	0.017
	G2	81 (22.1%)	56 (15.3%)	
	G3	96 (26.2%)	123 (33.6%)	

Anatomic neoplasm subdivision, *n* (%)	Fundus/body	62 (17.2%)	68 (18.8%)	0.014
	Cardia/proximal	23 (6.4%)	25 (6.9%)	
	Gastroesophageal junction	29 (8%)	12 (3.3%)	
	Antrum/distal	64 (17.7%)	74 (20.5%)	
	Other	4 (1.1%)	0 (0%)	

Reflux history, *n* (%)	No	88 (41.1%)	87 (40.7%)	1.000
	Yes	20 (9.3%)	19 (8.9%)	

Anti-reflux treatment, *n* (%)	No	71 (39.7%)	71 (39.7%)	0.257
	Yes	14 (7.8%)	23 (12.8%)	

*H*. *pylori* infection, *n* (%)	No	88 (54%)	57 (35%)	0.050
	Yes	6 (3.7%)	12 (7.4%)	

Barrett's oesophagus, *n* (%)	No	106 (51%)	87 (41.8%)	0.911
	Yes	9 (4.3%)	6 (2.9%)	

Age, median (IQR)		68 (59, 74.25)	66 (57, 72)	0.075

## Data Availability

All experimental data used to support the findings of this study are available from the corresponding author upon request.
